# Supplementary material for: Calnexin Is Involved in Forskolin-Induced Syncytialization in Cytotrophoblast Model BeWo Cells
Source: Biomolecules. 2022 Jul 28;12(8):1050. doi: 10.3390/biom12081050 (PMC9405722; doi:10.3390/biom12081050)
Supplement: Supplementary file 1 [file biomolecules-12-01050-s001.zip › biomolecules-1775400-supplementary.pdf]

Supplementary material

Title

Calnexin is involved in forskolin-induced syncytialization in cytotrophoblast model BeWo cells

Author Names

Hitomi Matsukawa[1], Midori Ikezaki[2], Kaho Nishioka[1], Naoyuki Iwahashi[1], Masakazu Fujimoto[3], Kazuchika Nishitsuji[2], Yoshito Ihara[2, *], Kazuhiko Ino[1]

Affiliations

[1]Department of Obstetrics and Gynecology, School of Medicine, Wakayama Medical University, Wakayama 641-8509, Japan

[2]Department of Biochemistry, School of Medicine, Wakayama Medical University, Wakayama 641-8509, Japan

[3]Department of Diagnostic Pathology, Kyoto University, Kyoto 606-8507, Japan

Corresponding author: Department of Biochemistry, School of Medicine, Wakayama Medical University, Wakayama 641-8509, Japan.

Phone: 81-73-441-0628. *E-mail address:* y-ihara@wakayama-med.ac.jp (Y. Ihara).

Supplementary Figure S1.

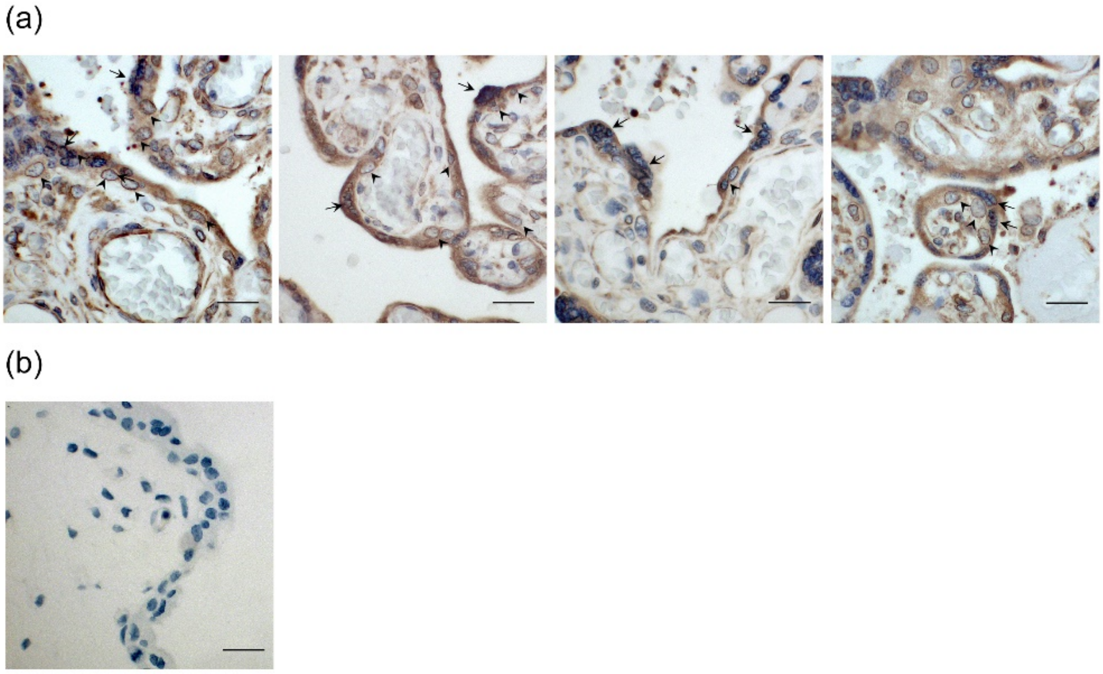

Supplementary Figure S1 Immunohistochemical analysis of CNX expression in third-trimester normal placentas.

(a) Additional representative images of third-trimester normal placentas that were stained with anti-CNX antibody. Arrows show CNX-positive STBs and arrowheads show CNX-positive CTBs. Scale bar, 20 µm. (b) Third-trimester normal placenta section was stained with an isotype control IgG. Scale bar, 20 µm.

Supplementary Figure S2.

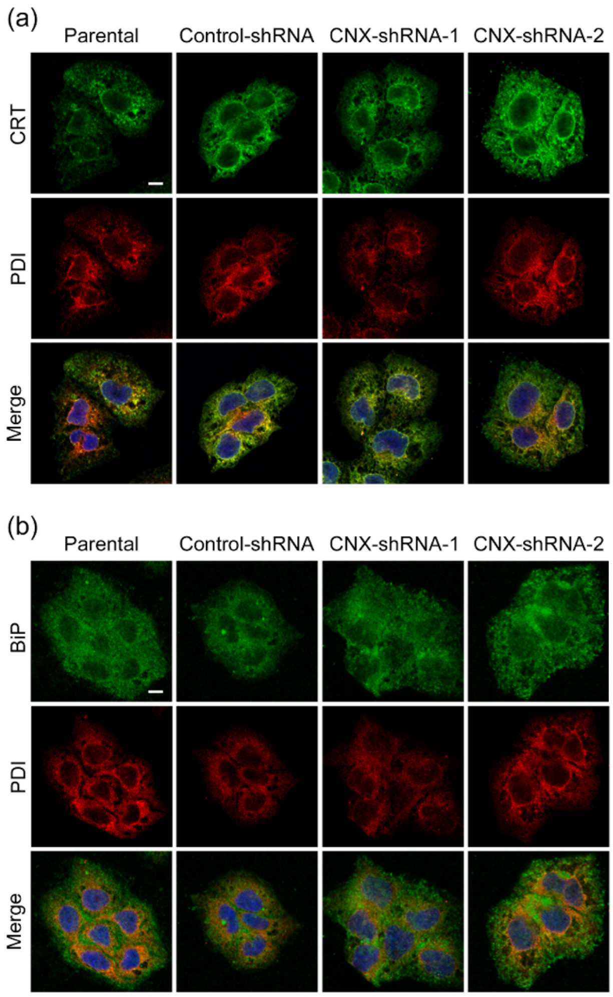

Supplementary Figure S2 Effect of CNX knockdown on intracellular localization of ER-resident proteins in BeWo cells.

Intracellular localization of CRT and PDI (a) and BiP and PDI (b) in parental, mock-transfected (Control-shRNA), and CNX-knockdown (CNX-shRNA-1 and CNX-shRNA-2) cells was investigated by using immunofluorescence microscopy with specific antibodies. PDI staining was used to indicate ER localization. Scale bar: 20 μm.

Supplementary Figure S3.

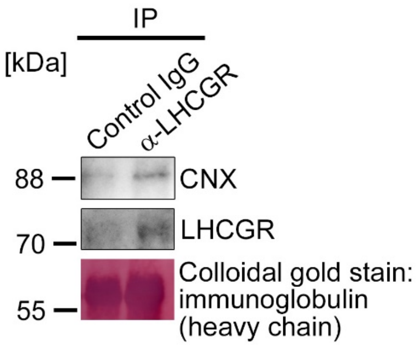

Supplementary Figure S3 Detection of the interaction between CNX and LHCGR in BeWo cells.

BeWo cells were lysed and crosslinked for 30 min on ice in the lysis buffer (PBS, 0.5% NP-40) containing 0.12 mM dithiobis(succinimidyl propionate). The crosslinking reaction was quenched by adding glycine to the final concentration of 9 mM and incubating for 10min on ice. LHCGR was immunoprecipitated with a rabbit polyclonal anti-LHGR antibody at 4°C for 2 h followed by an additional 1 h of incubation with protein G-Sepharose beads. Immunoprecipitated proteins were then eluted from the beads by heating at 95°C for 5min in SDS sample buffer, and samples were analyzed by means of immunoblotting with the rabbit polyclonal anti-LHCGR antibody or a rabbit polyclonal anti-CNX antibody.

Supplementary Figure S4.

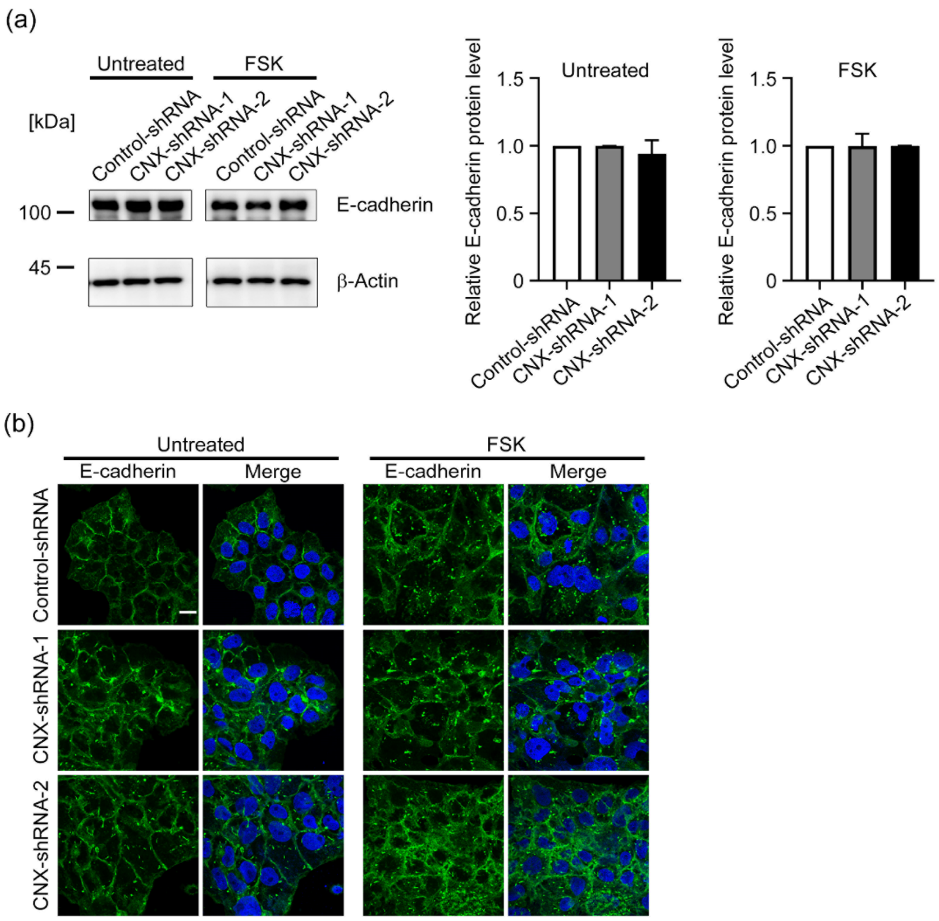

Supplementary Figure S4 Effect of CNX knockdown on E-cadherin expression in BeWo cells treated with FSK.

(a) Control-shRNA cells and CNX-knockdown cells (CNX-shRNA-1 and CNX-shRNA-2) were treated with FSK (50 μM) for 48 h, after which cell lysates were prepared and subjected to immunoblot analysis with an anti-E-cadherin antibody. The graphs show relative levels of E-cadherin. Data are shown as means ± SD of three independent experiments. (b) Control-shRNA cells and CNX-knockdown cells (CNX-shRNA-1 and CNX-shRNA-2) were treated with FSK (50 μM) for 48 h, after

which intracellular localization of E-cadherin was analyzed by using immunofluorescence microscopy with the anti-E-cadherin antibody. Scale bar: 20 μm.

Supplementary Figure S5.

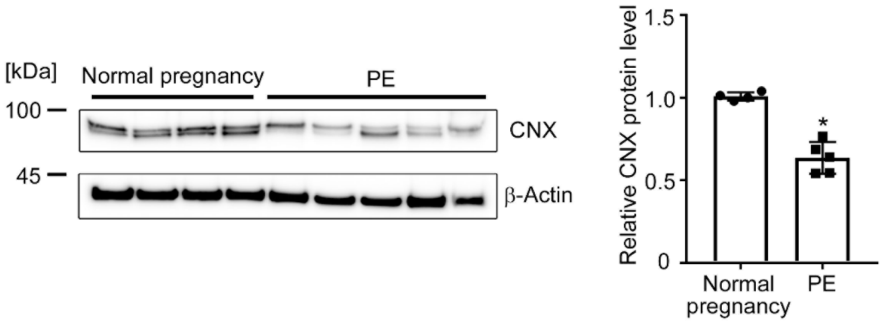

Supplementary Figure S5 CNX expression in human placentas.

Immunoblot analysis with an antibody against CNX in placental tissues from patients with PE (at 34–40 weeks) and placental tissues from women with normal pregnancies (at 34–39 weeks). CNX protein appeared as doublet bands. The band intensity was quantified by using densitometry and was statistically analyzed as described below in Materials and methods. β-Actin was used as a loading control. The graph shows the relative expression of CNX in human placentas. Data are means ± SD. *$p < 0.05$.

Supplementary Figure S6.

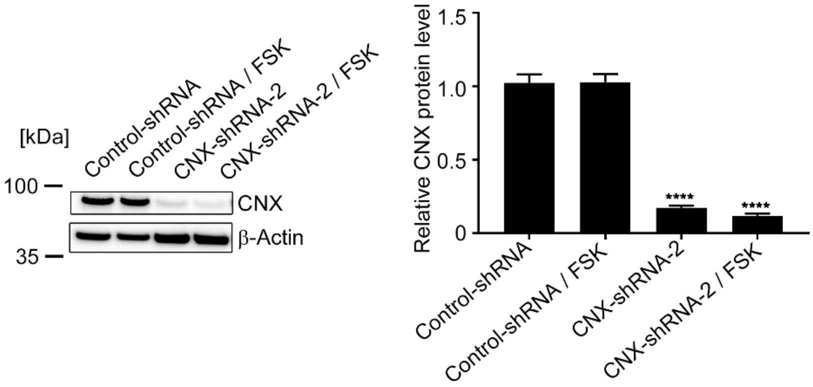

Supplementary Figure S6 Effect of FSK treatment on CNX protein levels.

BeWo cells and their CNX knock-down variant were treated with FSK (50 µM) for 72 h and protein levels of CNX were analyzed by means of Western blotting. The graph shows quantification of CNX levels. ****p < 0.0001 vs “Control-shRNA” by Dunnett’s multiple comparisons test.

Supplementary Table S1 Clinical characteristics of the study population.

	**Normal pregnancy**	**Preeclampsia**	***p* Value**
Characteristic	(n = 4)	(n = 5)	
Maternal age (years)	25.3 ± 5.5	37.0 ± 10.3	ns
Systolic blood pressure (mmHg)	118.8 ± 11.9	168.4 ± 9.5	< 0.0001
Diastolic blood pressure (mmHg)	74.3 ± 4.9	95.4 ± 9.2	< 0.001
Gestational age at delivery (weeks)	36.8 ± 2.1	37.0 ± 2.2	ns
Neonatal weight (g)	2579.5 ± 626.1	2559.4 ± 663.6	ns

n.s., not significant.

Materials and methods

Immunoblot

Cells were harvested and lysed in Lysis Buffer A (10 mM Tris-HCl [pH 7.5], 150 mM NaCl, 1% Nonidet P-40) containig 4 mM Pefabloc, 1 μM pepstatin, 1 μM leupeptin, and 200 μM phenylmethylsulfonyl fluoride (Roche, Basel, Switzerland). Lysates were centrifuged at 10 000×*g* for 10 min at 4 °C, and the supernatants were separated by 10% sodium dodecyl sulfate (SDS)-polyacrylamide gel electrophoresis. Proteins were then transferred to polyvinylidene difluoride membranes (Immobilon-P, Merck Millipore, Burlington, MA, USA). Membranes were blocked with 5% skim milk (BD Biosciences) and 0.05% Tween 20 (Wako Pure Chemicals) in Tris-buffered saline (pH 7.6) and were incubated with the following primary antibodies: anti-CNX antibody (N-terminal), 1:5000 (Cat No. ADI-SPA-865, Enzo Life Sciences, New York, NY, USA); anti-β-actin antibody, 1:1000 (Cat No. sc-1616, Santa Cruz Biotechnology, Dallas, TX, USA); anti-E-cadherin antibody, 1:3000 (Cat No. 610181, BD Biosciences, Franklin Lakes, NJ, USA), and rabbit polyclonal anti-LHCGR antibody, 1:2000 (Cat No. PA5-97923, Thermo Fisher Scientific, Waltham, MA, USA), followed by incubation with HRP-conjugated secondary antibodies (Cat No. P0448 and P0260, Dako, Glostrup, Denmark). Signals were identified by using the Immobilon Western Chemiluminescent HRP substrate (Merck Millipore) and were quantified means of by densitometry with ImageJ version 1.52a (National Institutes of Health, Bethesda, MD). β-Actin was used as a loading control.

Immunoprecipitation

BeWo cells grown overnight were lysed and crosslinked for 30 min on ice in the lysis buffer (PBS, 0.5% NP-40) containing 0.12 mM dithiobis(succinimidyl propionate), a membrane-permeable crosslinker, after which the crosslinking reaction was quenched by adding glycine to the final concentration of 9 mM. After 10-min incubation on ice, the samples were centrifuged at 3000 g for 5 min at 4°C. Immunoprecipitation (IP) was performed by incubating lysates with the rabbit polyclonal anti-LHGR antibody (1:200) or an isotype control rabbit IgG (Thermo Fisher Scientific) at 4°C for 2 h followed by an additional 1 h of incubation with protein G-Sepharose beads. Proteins were eluted from the beads by heated at 95°C for 5min in SDS sample buffer (0.125 M Tris-HCl, 4% [wt/vol] SDS, 20% [vol/vol] glycerol, and 0.01% [wt/vol] bromophenol blue and 10 mM DTT). The samples then underwent immunoblot analysis with the rabbit polyclonal anti-LHCGR antibody (1:2000) or the rabbit polyclonal anti-CNX antibody (1:5000).

Immunofluorescence microscopy

Cells grown on coverslips were fixed with 4% paraformaldehyde for 20 min at room temperature, after which cells were blocked and permeabilized with the Animal Free Blocker containing 0.05% saponin for 20 min at room temperature. The cells were then incubated with primary antibodies (anti-E-cadherin antibody, 1:300; anti-calreticulin antibody 1: 300 (Cat No. SPA-600, Stressgen, San Diego, CA, USA); anti-BiP antibody, 1:100 (Cat No. sc-13968, Santa Cruz Biotechnology, Dallas, TX, USA); and anti-PDI antibody, 1:300 (Cat No. SPA-891, Stressgen)), followed by incubation with secondary antibodies conjugated to Alexa Fluor 488 or Alexa Fluor 555 (Thermo Fisher Scientific). Specimens were mounted with Vectashield Mounting Medium containing DAPI (Vector laboratories, Newark,

CA, USA) and examined with a LSM700 laser scanning confocal microscope (Carl Zeiss, Oberkochen, Germany).

Collection of human tissue samples and preparation of these samples

Individual patients gave their informed consent for use of the specimens of placentas. Samples of third-trimester human placentas were collected at cesarean sections that were performed before labor started. We defined PE as the presence of a blood pressure value of ≥ 140 / 90 mmHg after 20 weeks of gestation and the presence of proteinuria (≥ 300 mg protein / 24 h). We used the diagnostic criteria of the International Society for the Study of Hypertension in Pregnancy to determine whether patients with PE were eligible for inclusion in the study. We excluded patients with multiple pregnancies, fetal chromosomal abnormalities, or fetal anomalies. For the study of immunoblots, placental tissues from PE patients (at 34–40 weeks, $n = 5$) and placental tissues from gestational controls (at 34–39 weeks, $n = 4$) were immediately frozen in liquid nitrogen and were stored at −80 °C before they were used. The Ethics Committee of Wakayama Medical University approved this study (authorization number:1690).

We used a Teflon homogenizer (As One, Osaka, Japan) to homogenize 500-mg samples of human placental tissues in 0.4 ml of Radioimmunoprecipitation buffer (Sigma-Aldrich) that contained the following protease inhibitors: 4 mM Pefabloc, 1 µM pepstatin, 1 µM leupeptin, and 200 µM phenylmethylsulfonyl fluoride (Roche, Basel, Switzerland). We centrifuged the homogenates at 18 000×*g* for 10 min at 4 °C, and we collected the supernatants and separated them by means of 10%

sodium dodecyl sulfate (SDS)-polyacrylamide gel electrophoresis. Proteins were then transferred to polyvinylidene difluoride membranes (Immobilon-P, Merck Millipore). Membranes were blocked with 5% skim milk (BD Biosciences) and 0.05% Tween 20 (Wako Pure Chemicals) in Tris-buffered saline (pH 7.6) and were incubated with the following primary antibodies: anti-CNX antibody (N-terminal), 1:5000; anti-β-actin antibody, 1:1000; incubation with HRP-conjugated secondary antibodies followed. Signals were identified by using the Immobilon Western Chemiluminescent HRP substrate (Merck Millipore) and were quantified means of by densitometry with ImageJ version 1.52a (National Institutes of Health, Bethesda, MD). β-Actin was used as a loading control.

Immunohistochemical analysis

Paraffin-embedded blocks of placental tissues were cut into 3-μm-thick sections, de-paraffinized, and rehydrated, after which endogenous peroxidase activity was blocked with 0.3% hydrogen peroxide in methanol. For antigen retrieval, these sections were heat-ed in 1 mM EDTA (pH 8.0) in a pressure cooker for 10 min. The sections were then incubated with the rabbit polyclonal anti-CNX (C-terminal) antibody followed by incubation with the HRP-conjugated secondary antibody. Signals were detected by using the Histofine Simple Stain MAX PO reagent (Nichirei Biosciences, Tokyo, Japan) and 3,3′-diaminobenzidine as the substrate. Sections were also stained with purified rabbit IgG (Thermo Fisher Scientific) that served as an isotype control.

Statistical Analysis

Data were analyzed via the Mann-Whitney U-test by means of Prism software (Version 7.04, GraphPad Software, San Diego, CA, USA). p values of < 0.05 were said to be significant.
